# IoT-Based Waste Management System in Formal and Informal Public Areas in Mecca

**DOI:** 10.3390/ijerph192013066

**Published:** 2022-10-11

**Authors:** Nibras Abdullah, Ola A. Al-wesabi, Badiea Abdulkarem Mohammed, Zeyad Ghaleb Al-Mekhlafi, Meshari Alazmi, Mohammad Alsaffar, Mahmoud Baklizi, Putra Sumari

**Affiliations:** 1 Department of Computer Engineering, College of Computer Science and Engineering, University of Ha’il, Ha’il 81481, Saudi Arabia; 2 School of Computer Sciences, Universiti Sains Malaysia (USM), Penang 11800, Malaysia; 3 Faculty of Computer Science and Engineering, Hodeidah University, Hodeidah P.O. Box 3114, Yemen; 4 Department of Information and Computer Science, College of Computer Science and Engineering, University of Ha’il, Ha’il 81481, Saudi Arabia; 5 Computer Science/Network Department, Faculty of Information Technology, Al-Isra University, Amman 11622, Jordan

**Keywords:** urban, IoT, smart city, smart infrastructure, environmental logistics, public area

## Abstract

Urban areas worldwide are in the race to become smarter, and the Kingdom of Saudi Arabia (KSA) is no exception. Many of these have envisaged a chance to establish devoted municipal access networks to assist all kinds of city administration and preserve services needing data connectivity. Organizations unanimously concentrate on sustainability issues with key features of general trends, particularly the combination of the 3Rs (reduce waste, reuse and recycle resources). This paper demonstrates how the incorporation of the Internet of Things (IoT) with data access networks, geographic information systems and combinatorial optimization can contribute to enhancing cities’ administration systems. A waste-gathering approach based on supplying smart bins is introduced by using an IoT prototype embedded with sensors, which can read and convey bin volume data over the Internet. However, from another perspective, the population and residents’ attitudes directly affect the control of the waste management system. The conventional waste collection system does not cover all areas in the city. It works based on a planned scheme that is implemented by the authorized organization focused on specific popular and formal areas. The conventional system cannot observe a real-time update of the bin status to recognize whether the waste level condition is ‘full,’ ‘not full,’ or ‘empty.’ This paper uses IoT in the container and trucks that secure the overflow and separation of waste. Waste source locations and population density influence the volume of waste generation, especially waste food, as it has the highest amount of waste generation. The open public area and the small space location problems are solved by proposing different truck sizes based on the waste type. Each container is used for one type of waste, such as food, plastic and others, and uses the optimization algorithm to calculate and find the optimal route toward the full waste container. In this work, the situations in KSA are evaluated, and relevant aspects are explored. Issues relating to the sustainability of organic waste management are conceptually analyzed. A genetic-based optimization algorithm for waste collection transportation enhances the performance of waste-gathering truck management. The selected routes based on the volume status and free spaces of the smart bins are the most effective through those obtainable towards the urgent smart bin targets. The proposed system outperforms other systems by reducing the number of locations and smart bins that have to be visited by 46% for all waste types, whereas the conventional and existing systems have to visit all locations every day, resulting in high cost and consumption time.

## 1. Introduction

An enormous number of Muslims go to the Kingdom of Saudi Arabia (KSA) every year to perform Umrah and Pilgrimage (Hajj). Holy areas of worship are visited by millions. These areas are the holy mosques al Haremeyn (Makkah and Medina) and Al-Masha’ir (Arafat, Mina and Muzdalifah) [1]. In recent decades, the number of pilgrims visiting KSA for Hajj (the 12th month of the Islamic lunar calendar) has notably risen, with an annual increase of 1.15% from 1993 to 2014. This increase comes as a result of the continuing widening of the holy mosques, improving safety and transportation services, and minimizing aggregate time and cost [1,2,3]. The KSA makes approximately fifteen million tons of municipal solid waste (MSW) every year, with a daily rate of 1.4 kg per individual. The median rate of waste generation is predicted to grow to 30 million tons by 2033 due to persistent population growth and urban widening [4].

**Figure 1:**
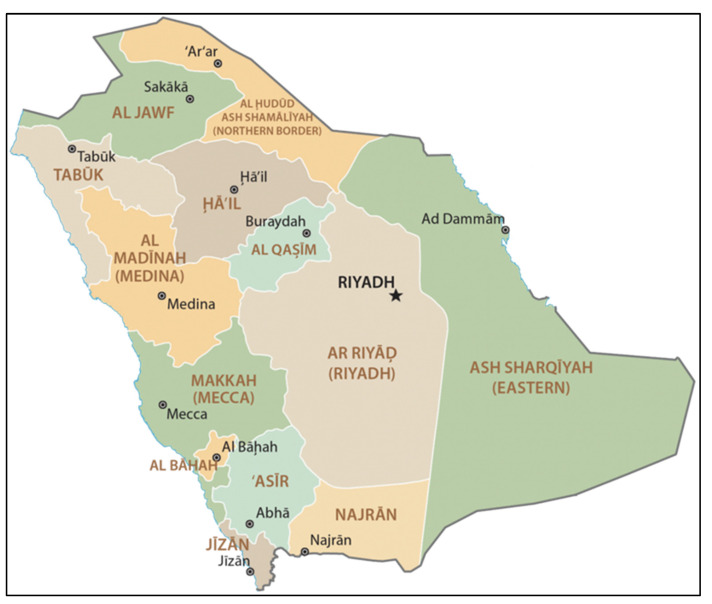
Saudi Arabia region map.

Regions or provinces are the top-level executive unit in KSA. Presently, 13 provinces in KSA involve the Northern Borders Region, Al-Jawf, Tabuk, Ha’il, Medina, Al-Qassim, Riyadh, Eastern, Mecca, Al-Bahah, ‘Asir, Jizan and Najran, as shown in Figure 1 [5].

Regions or provinces in KSA are separated into 136 governorates, which are the second-level executive units.

**Figure 2:**
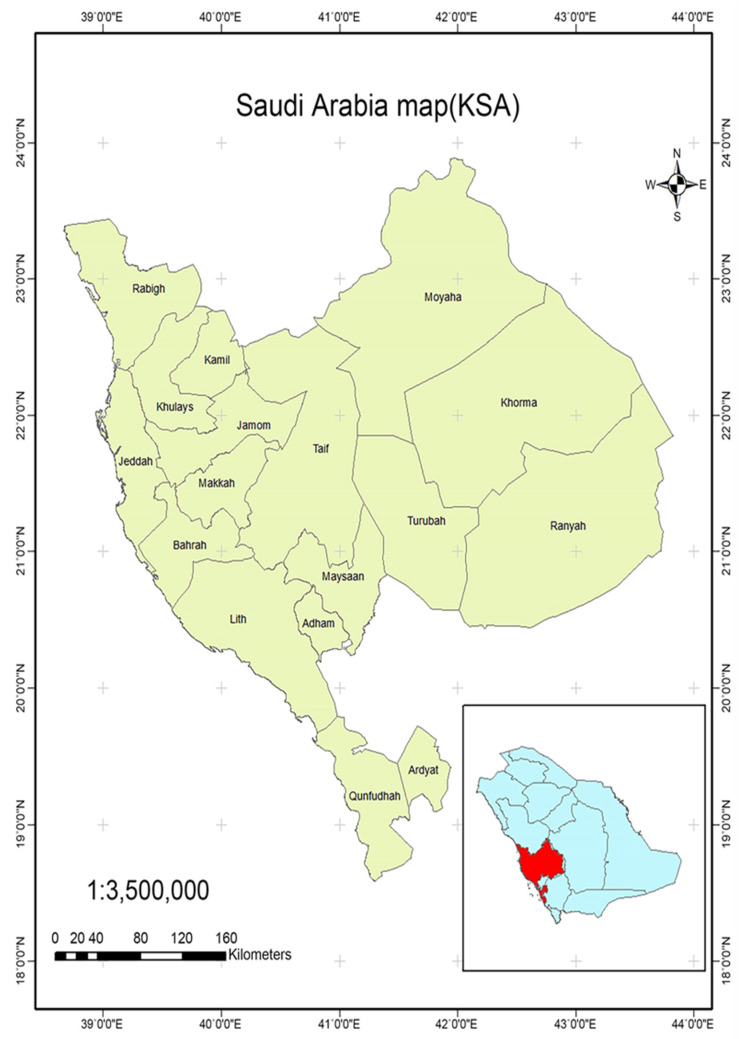
Mecca governorate map [8].

This paper is focused on Mecca (Makkah al-Mokarramah) as a case study. Mecca is divided into 17 governorates, including the central governorate of Makkah al-Mokarramah, as shown in Figure 2 [6,7].

**Figure 3:**
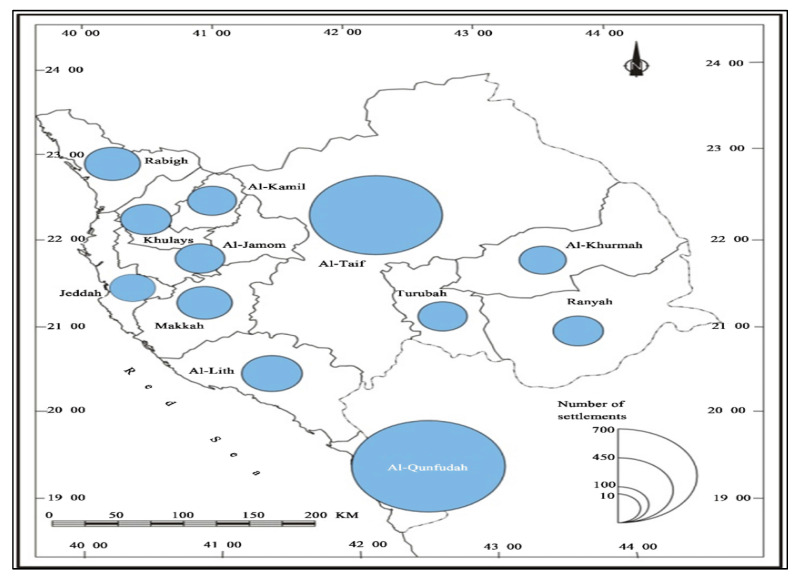
Holy Makkah al-Mukarramah municipalities [14].

The center of Makkah al-Mokarramah is the holy city of Makkah. Makkah al-Mokarramah is considered the largest in KSA, with an area of 153,148 km^2^, and includes 12 municipalities, as shown in Figure 3: Adham, Al-Jumum, Al-Kamil, Al-Khurmah, Al-Leith, Al-Qunfudah, Al-Taif, Jeddah, Khalis, Makkah, Rabigh, Rania and Trouba [6]. In 2017, the number of inhabitants of Makkah was 8,557,766, with a density of 56 people/km^2^ in 2017. Makkah has enormous inhabitants that result in the highest waste generation.

According to the study by Aina et al. (2013), the shape of the city of Mecca prior to modernization in KSA was recognized by a strong traditional Arab community pattern that emphasizes the importance of public and private spaces focused on Islamic manners. Typically, the urban area has expressed stability of social, religious, environmental and cultural values as it is the side of the form and structure of the city. Appropriate public spaces have been provided to accommodate social requirements and avert spatial redundancy and have been projected to adjust to the desert climate and challenging environment. Gradual modification, rather than large-scale development, was evident through the pre-modernization era in the mid-19th century. This vernacular urban style is known as a heritage of Arab architecture and design and characterizes the Islamic civilization and traditional urban settlements of Mecca [5,9].

As much as the population is increased, the waste accordingly is increased. From the data of the General Population and Housing Census 1431 AH (2010 AD), the population of the Mecca region is 6,915,006, whereas that of the district of Mecca is 1,578,722 [10,11]. According to the Department of Population and Vital Statistics authority in Holly Makkah Municipality [12], the average population growth rates for the Makkah region between 2010 AD and 2016 AD amounted to 2.806%. The total MSW received by the general landfill in Makkah amounted to more than 68 thousand tons during the period from 1 to 13 of the month of Dhu al-Hijjah 1442 AH. The Secretariat indicated that the landfill received 28,571 tons of organic waste (domestic—commercial), 38,081 tons of construction, demolition and rubble waste, and 1940 tons of agricultural waste, large-sized special waste and animal waste [12]. The total organic waste received by the general landfill in Makkah during the period from the 1st to the 5th day of Ramadan 1442 AH amounted to 12,348.7 tons [13].

On the other hand, the Al-Medina region produces around 887 thousand tons of MSW yearly. This rate will increase due to the increasing number of pilgrims every year. The highest average of waste production happens in the last 10 days of Ramadan (the 9th month of the Islamic lunar calendar) due to the enormous numbers of local and foreign pilgrims in the Makkah and Medina regions [1]. The KSA’s Vision 2030 strategy targets to decrease all kinds of waste and produce renewable energy from natural origins involving generated waste. The strategy has constructed a roadmap for the execution of a solid waste management system to improve the economic and environmental importance of waste behind reuse and recycling [15].

Related studies in smart cities, healthcare and waste management mostly concentrate on solid waste management, which covers various waste kinds as well as food waste. Internet of things (IoT) architecture is utilized in some studies to improve waste administration performance [16,17,18]. Study [4] proposed an integrated method for an organic waste management system to achieve sustainable OWM in the context of state policy and appropriate frameworks, suitable technology, institutional order, operational and monetary administration, and people consciousness and involvement. In line with the Integrated Approach to Achieve a Sustainable Organic Waste Management System in Saudi Arabia [4], this paper focuses on the organic waste source, especially food and medical waste. It also proposes a waste collection strategy to perform waste collection around the city, including the areas that cannot be reached by conventional trucks.

## 2. Background

This paper treats the said crucial issues of source waste production, gathering and transferring in Mecca in KSA by utilizing an efficient proposed waste management system.

### 2.1. Effect of Food Waste

At least two billion tons of MSW are created yearly, and approximately 33% of this value is considered environmentally harmful, based on the World Bank report in 2020. The mean value of waste generated per person every day is 0.74 kg according to the universal standard. These amounts range from 0.11 kg to 4.54 kg. Considering only 16% of the world’s citizens, high-income export represents about 34% or 683 million tons of worldwide waste [19,20]. In the last decades, waste production has extremely grown all over the globe with no signs of reducing. Universal MSW production is anticipated to be maximized by harshly 70% to 3.4 billion metric tons by 2050 due to some reasons, such as economic development, global population increase, consumer shopping behavior and civil extension. Human beings produce millions of tons of waste annually, and this is promptly becoming a universal issue. The need for countries to present convenient waste treatment and disposal services has turned ever further considerable with the enormous amount of waste stacking.

As stated by United Nations Environment Programme (UNEP), statistics are divided into unfair methods through state income classes and provinces. According to a regional scale, developed economies in Northern and Western Europe, North America, and Australasia have achieved the greatest improvement in statistics gathering in the areas of the household, food facilities, and retail. At the same time, many developing economies in Africa, the Caribbean and Latin America suffer from data insufficiency [21]. Moreover, the UNEP statistics stand against the traditional perspective that food waste is an issue that only affects sophisticated, high-income countries and rather spotlights it as a worldwide source of anxiety. Consequently, the extremely remarkable duty in decreasing the lack and waste of food is to be aware of the trouble. Thus, the population should recognize the extent of the trouble, the quantity of food wasted and the matter of food employed. However, the first procedure, and a form of prevention, is to evaluate the expense [21]. Although UNEP recognizes its data are lacking in terms of numbers, statistics are further plentiful in developed economies, implying that it still supplies linked information for the Financial Stability Institute. Household waste is habitually larger than the overall other food waste classifications (retail and food service waste). However, the US is excluded because of the predilection of the American nation and the culture of eating outside the residence.

**Figure 4:**
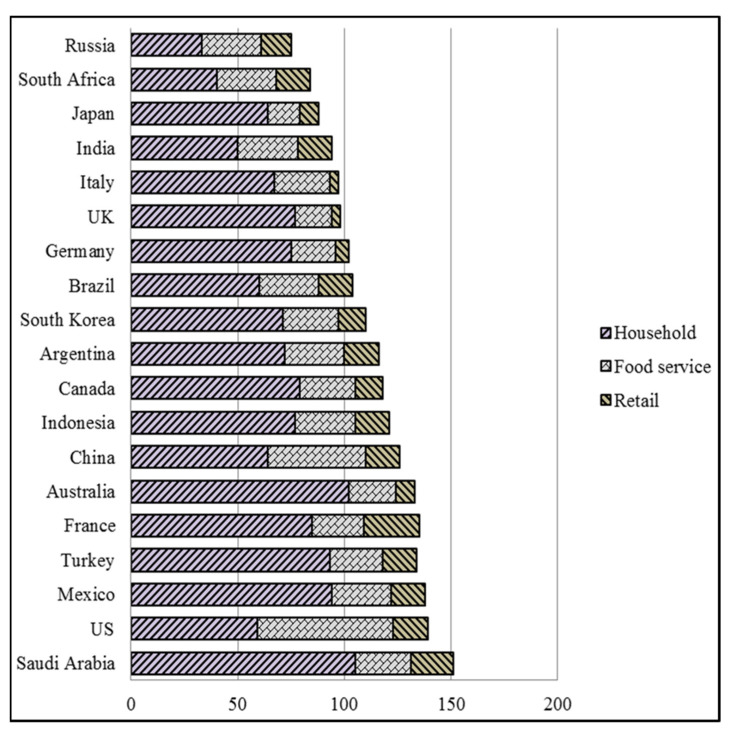
Food waste index flowchart (kg/head/year) 2021.

Group of Twenty or G20 is an intergovernmental forum of major, developed economies (the European Union and 19 countries) that has a crucial turn to proceed. The members of G20 perform 60% of inhabitants, 80% of economic manufacture and 75% of greenhouse gas (GHG). The effect of this commercial prosperity translates into environmental responsibility. The G20 countries that proceed well in all three categories are Japan, the UK, Germany and Italy. Japan ranks in the highest six for every three categories, whereas the UK and Germany have a mission to perform on decreasing household waste, as shown in Figure 4. On another side, KSA and France suffer extremely from household and retail waste. Moreover, Turkey and Mexico rate unsatisfactorily, as shown in Figure 4.

As pointed out previously, the earliest, largest religious assemblies worldwide occur yearly in KSA. Numerous Muslims from all countries perform Pilgrimage to esteem at Al-Haram (Holy Mosques in Makkah and Medina) and Al-Masha’ir (Arafat, Muzdalifah and Mina) [1]. The entire area of the Al-Haram mosque in Makkah is about 356,800 km^2^, which involves indoor and outdoor prayer spaces. Moreover, more than two million Muslims assemble to worship during Ramadan and Hajj [22]. Rubbish gathering and disposal are fundamental missions for those responsible during the Ramadan and Hajj seasons, which affect critical growth in waste generation in a small period of time. Furthermore, this attitude turns into further demand due to the aggregate of increasing plastic and food waste [23]. The majority of waste is disposed of in landfills that lack leachate and landfill gas assemblage [24,25]. This action produces water and soil pollution and releases massive amounts of GHGs [26]. Consequently, the requirement for a timely waste management system in the Makkah region has concentrated on dealing with the large amounts of waste created by the inhabitants and pilgrims [1].

Rubbish is fetched employing private or community grants and disposed of in landfills in KSA. The waste management system in KSA stands in need of waste disposal facilities and transfer burdens. In the upcoming decades, the majority of landfills are anticipated to be overcrowded and incompetent to be employed. Although the retrieval of energy, recycling, and reusing are generally thought about carefully, they are still at the initial stage. Recycling and classification of waste are done by an unofficial efficient section. Mostly, the average recycling varies from 10% to 15% because of the entity of unofficial sections that reuse plastics, minerals and papers from household waste [27].

The current gathering system in populated civic areas grows the charge of placing air purification devices in residential sectors. In addition, the small position of trucks and waste containers brings about a problem during the procedure of the gathering method, mostly in historical zones as well as the informal and formal generic regions. The assembly of every category of waste that depends on a pre-established track causes dispensable costs and insufficiency of outfits. In many situations, unfilled bins are usually gathered first, whereas full bins spill over, which results in health dangers, rising cleaning expenses and complaints from inhabitants.

This paper concentrates on a structured waste management system that relies on physical IoT devices to enhance waste-gathering in various locations and considers the main step for recycling waste in Mecca as a case study. The improvement in cloud facility employment, databases and implementations will connect softly through various physical IoT devices, imposing new links between the existing and new systems. Moreover, the ensuing data networks will reduce costs and risks and improve the waste management process. The proposed planning, the utilization of gathered data distributed and linked between bins, vehicles and smart containers, as well as the inhabitants’ information and other public activities, permit automation and coordination of the spotting of the waste sector and waste bins for recycling and reusing. Hence, minimizing waste-gathering costs in KSA is anticipated. The execution of the upcoming Internet technology developed by the implementation of the Internet Protocol on several wireless sensors follows the IoT model. Numerous sensor devices can cerebrate to be components of wireless sensor networks (WSNs) when used in an urban area. These sensors gather and handle continuous ambient data to make efficient legacy city infrastructure, which is introduced as smart cities [28].

### 2.2. Waste Management System in Mecca

The current system in Makkah was established in 2011 by Mari Matic. Utilizing the Metro Taifun System, waste is collected from 318 waste access points and then carried through a 30-km pipe network to a Central Utility Complex [29]. The schemes of the project are as follows:

1. Waste is transferred in underground pipes with a range of 30 km beneath the central zone of Makkah;
2. It is expanded to 7 km outside the Haram domain;
3. The project permits the moving of 600 tons per day in more than 318 locations.

Researchers in [30] presented a smart waste management system called Makkah-SWMS to increase the performance of the existing system by using sensor networks and recycling concepts in a proposed smart architecture. The system architecture includes three levels: physical, middleware and management [30].

The physical level shows the infrastructure that includes the trash bins with sensors that have access to the Internet. The middleware includes IoT and the management system point. Finally, the management level focuses on smart landfill, smart trucks and smartphones. The proposed system combines and transmits the data from smart trash bins using WSN to the main system for more procedures. The gathered data are used to observe and enhance the daily picking of trash bins when they reach the maximum capacity. Utilizing GPS information, the trucks receive the correct direction [30]. According to [30,31], more than half ration of the waste quantities in Makkah come from food. However, looking at the problem of the waste management system from another perspective, the population and residents’ attitudes directly influence the control of the waste management system.

The target of the study in [5] was treated by looking into urban growth in Makkah, specifically the Al-Khalidyah district, to realize the evolution of public areas in Makkah and the scale of facilities provided to local inhabitants according to inhabitants’ opinions on facilities provided in formal and informal settlements. Characterizing the philosophical scenario of a research study is essential due to its effects on the research method and results [31,32,33,34,35,36]. However, the selected study model constitutes the procedural structure that is utilized to realize a particular social phenomenon. Hence, the case study scheme provides a justifiable approach in the current research because it gives an appropriate perception of a phenomenon in its natural framework [36,37,38]. The case study scheme, in specific, enables conducting comprehensive research in Al-Khalidyah by evaluating the public area and the level of facilities presented to inhabitants at the district scale. This scheme involves the gathering of various origins of data, which helps improve the capability to describe specific procedures and results.

The case study scheme is utilized in various domains involving sociology and cultural studies. It is employed to look into people, communities or attitudes at a specific area and time. Case studies perhaps depend just on qualitative data-collecting techniques or on a combination of methods. Public space is closely linked to waste administration and civilized excellence in the case of Mecca. The kinds of public spaces denote various kinds of waste administration and consider the broader feature and socio-economic conditions of the area. The formal waste management system covers formal and informal settlements that can be reached through municipal services. By contrast, such services do not cover settlements located on rough terrain with narrow roads and lanes. Case studies also confirm the multiple functions of public spaces, whether generated via formal design or generated naturally by users. The use of public spaces indicates the various requirements of users in different age categories and may happen regularly or irregularly. In addition, public spaces in both localities show that waste management is to a lesser extent due to the primary type of waste administration (no sorting), reaching waste sources and availability of waste collection services [5].

**Figure 5:**
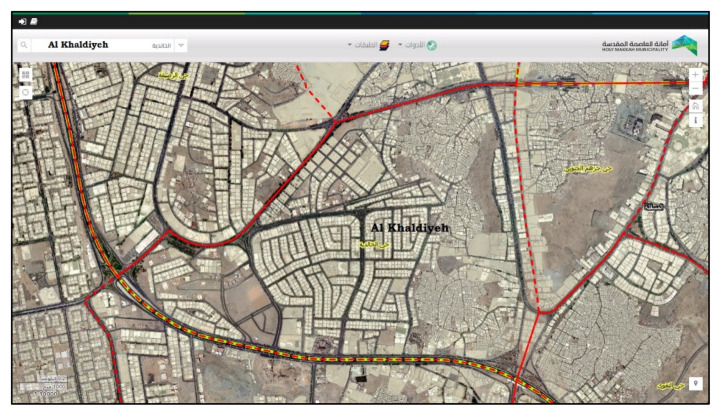
Khalidiyah district center. Source: https://maps.holymakkah.gov.sa/ (accessed on 6 October 2022).

Approximately 53 open public spaces are in the Khalidiyah area, or approximately 19% of the entire area, as shown in Figure 5 [5]. Three main kinds of open public spaces can be distinguished: formal gardens and parks, multifunctional roads and nongovernment land. However, four kinds of open space exist if unutilized, deserted private land is considered. In a general view, formal parks are well planned and supply various features for inhabitants and guests, involving linking walkways, shade and seating areas, children’s play areas, and drink and ice cream carts. Moreover, informal open spaces are used for public relaxation, car washing, exercise and public communication.

A formal park is available 24 h a day, at different times and is used in different ways. In terms of solid waste in the park, solid waste includes boxes and cans of food, and plastic, with garbage bins full in the morning and require regular cleaning. Trash cans are available, but they are not enough to dispose of trash because they are overflowing, and trash is laying on the ground, in addition to the other waste resource places in Khalidiyah, as shown in Table 1.

In portions of the Khalidiyah area, deserted and unutilized private and public land is informally used as public parking lots and playgrounds. This area has no garbage collection services. Some deserted private land is used by mobile vendors to sell food as well. Landfills are closely common in informal communities, residing unoccupied land with well reach to residential properties and open areas. Residents, mainly foreigners, utilize this area for meetings, children’s games and general communication. Garbage is dumped and scattered throughout the region. This state of affairs is conducted on cases of inefficiencies in the administration of public facilities in the urban area, especially in supplying public areas and solid waste administration for inhabitants. According to this case, the objective of this paper is to propose an IoT-based efficient waste management system for public areas in Macca. The proposed work focuses on the scale of facilities provided to domestic residents according to the resident population in Macca.

One of the problems often faced by researchers in the field of local solid waste management is the lack of documented data on the quantity and quality of waste from the authorities, as well as its characteristics or types. Most current studies on MSW in the KSA rely on different sources in their treatment of waste issues, according to the main objectives of the paper, as no official site periodically provides data with scientific classification. In a smart city, the existing waste management system is transformed into a smart system via the IoT concept. Several studies have attempted to develop an intelligent system for real-time, low-cost waste collection [39,40,41,42,43,44,45,46,47,48]. The following literature review focuses on current research using IoT concepts to implement intelligent solid waste management systems in Mecca.

The authors [49] presented algorithms to control the adjustment between the instant gathering and its expense. The proposed method decreases the demanded time for serving excessive-precedence regions with maintaining the anticipated overall achievement at a top level. Extensive simulations prove the proposed schemes on each qualitative and quantitative standard that can be followed to investigate their strengths and weaknesses. The usage of IoT structure plays an important role in optimizing waste control within the context of smart cities [50]. The typology usage of a sensor node is supplied with a single-chip microcontroller, and the sensor is capable of knowing the degree of packing trash containers through the usage of ultrasounds. The information dispatch unit is primarily dependent on low-power wide area network technology. The analyzed node structure considers energy-saving techniques that reduce energy consumption to extend the lifetime of the batteries by optimizing the hardware and software program. In a study [51], the author proposed smart garbage and waste-gathering bin scheme to avert overloaded rubbish containers located in public areas. The bins were implemented with a microcontroller scheme with infrared (IR) wireless structures that show the present case of bins on a mobile browser with an HTML page.

The idea of utilizing a camera was proposed in the study [52]. The authors presented a decision support system for effective waste gathering in smart cities. This system involves a scheme for information sharing amongst truck drivers in real-time to optimize the performance of the dynamic route of waste gathering. Surveillance cameras are included for capturing the difficult zones and offering proof to the authorities.

However, the comparison between a load of waste during the month of Ramadan and Hajj with the usual days increases from 122% to149%. Moreover, the average of increasing in the month of Hajj compared with the other months ranges from 242% to 304%. Thus, making improvements in the role of public-private partnerships is important to enhance the MSW in the Makkah zone, which involves the reusing and recycling of waste. Theoretically, it can anticipate that weather may be stored from 5.6 thousand tons of emission of methane (CH4). Methane is also known as GHG, so its existence in the atmosphere affects the climate system and the globe’s temperature. Moreover, 140.1 thousand Mt CO2 eq. of global warming potential has a carbon credit income value of 67.6 million Saudi Arabian Riyal (SAR) through recycling aluminum, metals, cardboard and glass. Thus, through effective recycling, a net revenue of 113 million SAR can be added to the country-wide financial system yearly, most effective from Makkah Province [3].

The authors in [30] presented a smart waste management system named Makkah-SWMS. This system includes two stages. In the first stage, it separates the waste into five portions and transmits a notification message to the monitoring system when the trash bin is overloaded. The sensors in the trash bin function as follow:

- In the first level, the capacitive nearness sensors split up papers and plastic into the trash bin, then check the papers and move them to part A whilst the plastic is transferred directly to Part B;
- In the second level, the metal sensor is utilized to check metal and transmit it to part C;
- In the third level, the IR sensor checks the glass and moves it to part D;
- In the fourth level, the remaining waste is moved to part E;
- Lastly, the ultrasonic sensor observes the scale of the trash bin fill.

**Figure 6:**
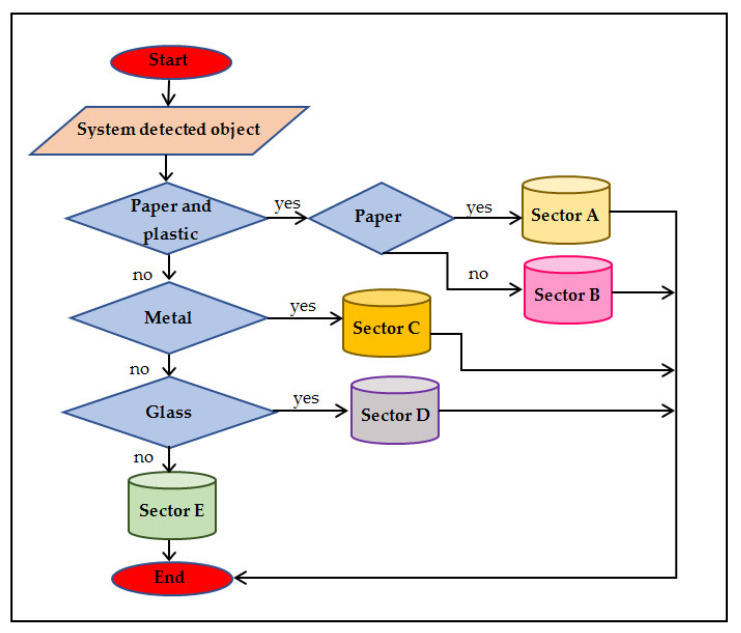
The architecture of Makkah-SWMS [29].

In the second stage, when the trash bin is overloaded, an SMS is sent to the waste garbage trucks by radio frequency receiver by utilizing the Arduino IDE system through GSM/GPRS. Figure 6 shows the flowchart of the operation of the sensors in the system.

The ultrasonic sensor is located at the head of the trash bin. It is utilized to sense the scale of the waste fill. The ultrasonic sensor declares whether the trash bin is full and transmits the radio frequency signal to the system control. This signal is received through a radio frequency receiver that is linked to the system control. However, the paper highlights that food waste achieves more than half a percent of the types of waste, and the proposed system does not consider food a waste category part.

The study in [53] proposed a system to enable municipal waste management to observe the waste scale case inside the trash bin. The system includes the following: (i) the design of sensor circuitry, (ii) the updated database and (iii) mobile application (Apps). The sensor circuit is established with a microcontroller interface as well as an ultrasonic sensor to calculate the waste scale in the trash bin. The data collected by the Wi-Fi module are saved in the database. The data in the database are transmitted to the mobile application (Apps) whenever the employee accesses the application.

In contrast to the previous studies, smart cities are densely populated and urbanized. Thus, waste is difficult to gather utilizing normal trucks, mostly within peak hours. However, the kind of waste decides the volume of the trucks. Hence, small trucks minimize traffic and facilitate moving around the urban area. Moreover, this paper is concerned with all the public areas around the region, including the informal areas that are not considered in the existing works, as mentioned earlier.

The next section presents the proposed system in three phases, which focus on trash bin distribution according to the waste generation in each area as the first phase. The waste generation is based on the population density in that area and other waste source locations. Then, the waste collection strategy utilizes IoT technology to improve waste gathering and provide information for statistical analysis. The last phase is the optimization of waste collection transportation.

## 3. Proposed Design

**Figure 7:**
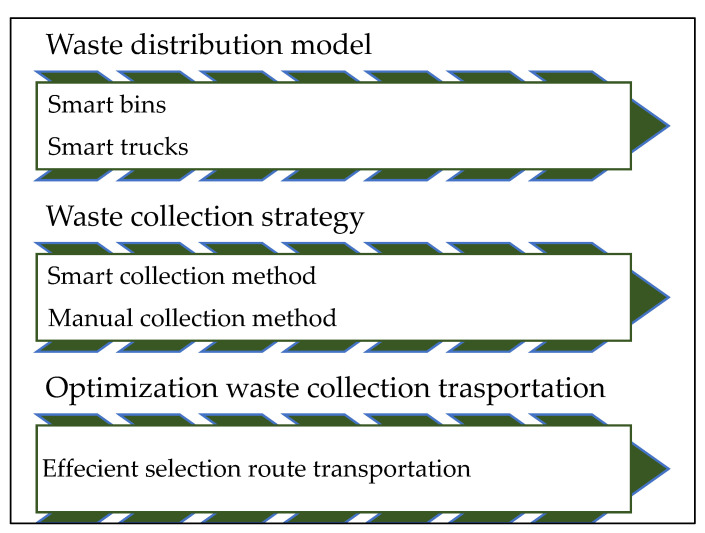
Waste management system phases.

The proposed IoT-based efficient waste management system of public space in KSA includes three phases, as presented in Figure 7. The first phase is the waste distribution model of waste tolls amongst the city based on people distribution and waste resources place. The second phase is the waste collection strategy that uses IoT technology by providing smart bins that are used to improve waste gathering and provide information for statistical analysis. The third phase is the optimization of waste collection transportation. Smart trucks of different sizes are used to improve the transportation of gathered waste.

**Figure 8:**
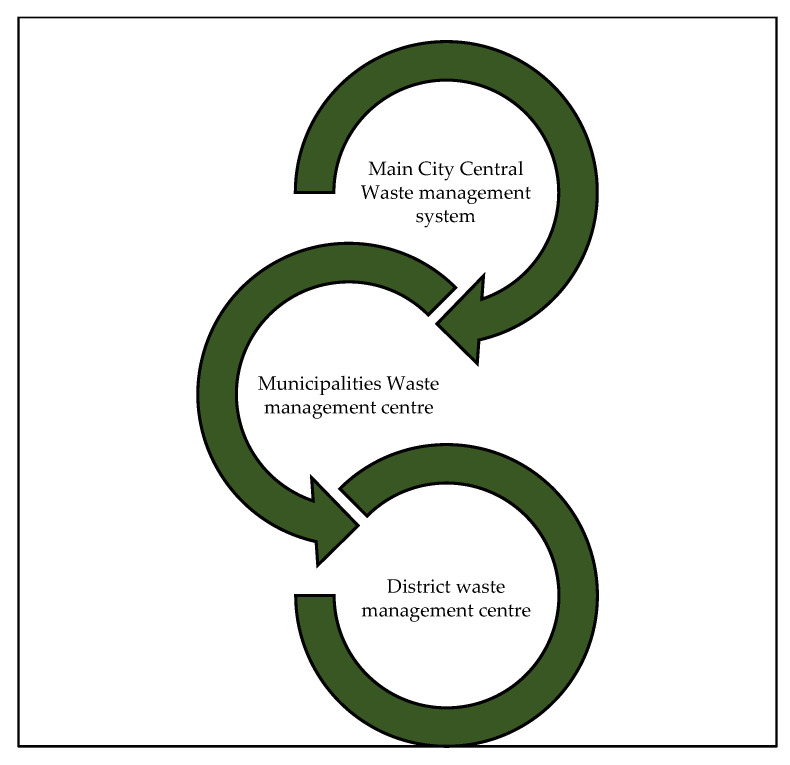
Waste management system administrative centers.

The architecture presented in this paper consists of three levels: Main City Central Waste Management (MCCWM), Municipalities Waste Management Centre (MWMC) and District Waste Management Centre (DWMC), as shown in Figure 8. MCCWM processes and manages all the waste system information for the city that is received from the second level, that is, MWMC for the municipalities around the city. Each MWMC is responsible for a different number of districts and manages the entire district’s waste management procedures through the DWMC. The Waste Management System administrative centers are responsible for managing the information and providing government statistic reports. The information according to the structure level includes the amount of waste volume collected per day based on the waste type or district or municipality and the number of collection times for each location.

**Figure 9:**
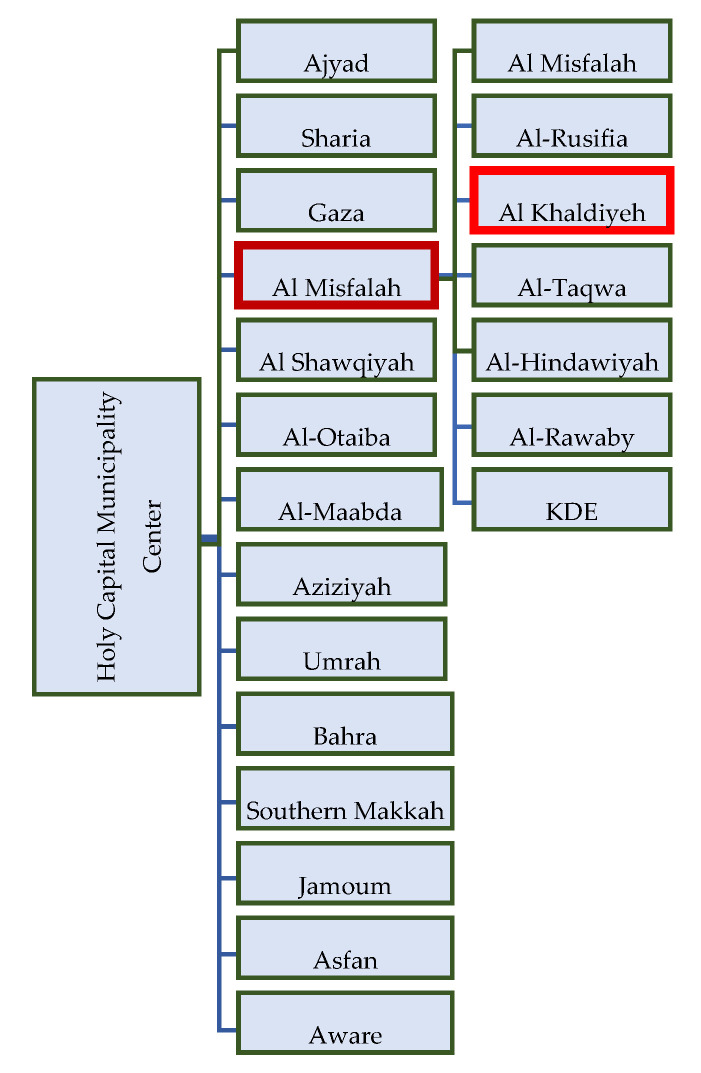
Waste management system distribution.

Figure 9 presents the district of Al Khalidiyeah as part of DWMC as a case study under the Al Misfalah municipality (MWMC) under Holy Capital Municipality Centre (MCCWM).

### 3.1. Waste Distribution Model

This phase is demonstrated by MCCWM, in which geographical information and demographic information are provided. All government resources are available in the central city. Based on the population of each municipality around the city and the density of districts in each municipality, MCCWM estimates and provides an adequate amount of equipment, including waste collection trucks and trash bins for each municipality. MWMC also calculates the required amount of equipment for each district based on the waste source locations. Examples of waste source sites are homes, supermarkets, groceries, bakeries, restaurants and others, as previously presented in Table 1.

**Figure 10:**
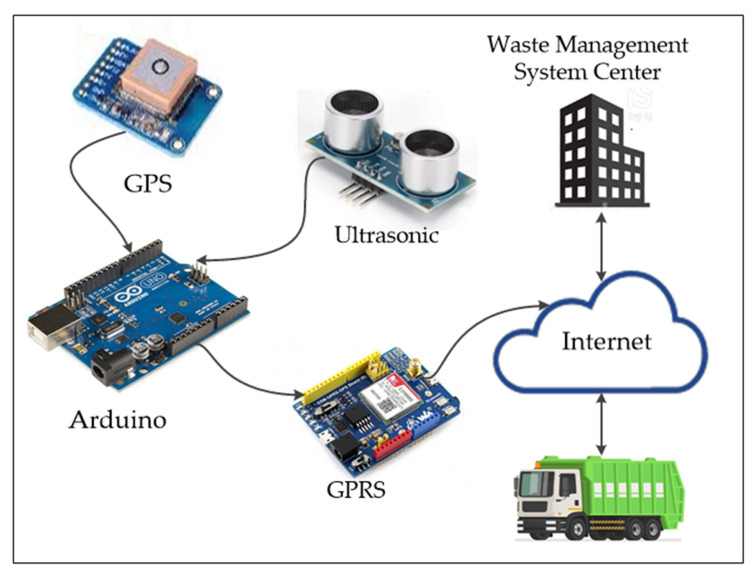
Integrated technology devices/applications.

A smart bin is proposed in this paper to support waste collection and provide a healthy environment. Each smart bin and truck includes the following gadgets: ultrasonic, Arduino, GPRS and GPS connected through the cloud server. In addition, the application is used for monitoring and interacting with the system after the action has been done. Figure 10 presents an example of small gadgets/devices together with a web-based system that permits the combination of the IoT as a new-generation technology.

The system plans to test the previously available waste management system and gather the information to perform a more optimized waste management system. The system not only informs about the smart bin scale but also notifies the worker working on it. It will assist domestic corporations, government organizations as well as people who still utilize manual ways of gathering garbage from trash bins.

However, as shown previously, households in KSA generate more waste food compared with food services and retail than in other countries, referencing that it should pay more concern to all the resident’s service locations, including the open public area and the historical area.

### 3.2. Waste Collection Strategy

#### 3.2.1. Smart Collection Method

In the waste collection stage, DWMC receives all the waste information from the smart bins by using GSM or GPRS via the cloud system in accordance with the area’s infrastructure. The smart bin sends an urgent message to DWMC to request a truck to collect the waste only if the smart bin level is full and attains its threshold weight. The threshold residual free space defined the smart bin level status as full when it reaches 90% of waste volume. DWMC has all the required data about trucks, bins and containers. Based on the status of smart bins and waste type, the efficient route is calculated from the central location of the active trucks towards the urgent smart bin location. Then, the task of collection is assigned to the available, active smart truck. The smart truck receives the notification based on the location, available free space and waste type. The system shows the truck close to the target bin location. Table 2 presents the parameters that represent the smart bin waste information and its status.

**Figure 11:**
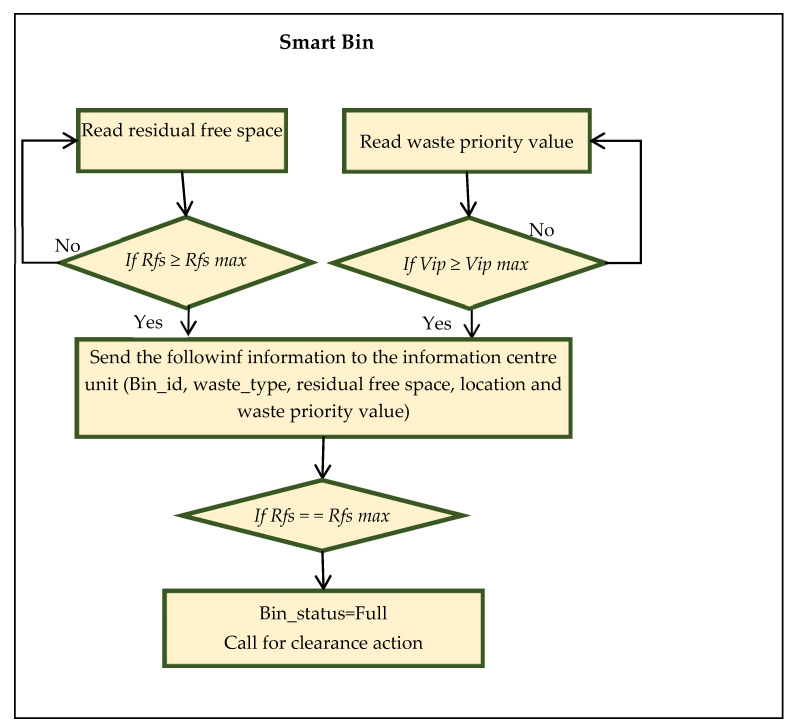
Intelligent smart bin handling information.

The proposed system utilizes the intelligent routing algorithm to find the most efficient route to collect the waste. Figure 11 presents the flowchart of processing information in the smart bin based on the following conditions:

If Rfs ≤ *Rfs max*

  {

    Bin status = Full

    Send the urgent notification

    Perform an action

  }

  else

  {

    Read the residual free space;

    Update information

  }

if Vip ≥ Vip max

  {

    Send the information

    Perform an action; 

    Update information

  }

where:

*Rfs* denotes the residual available space.

*Rfs max* denotes the maximum threshold value of residual available space.

*Vip* is used for the weight measurement of the danger level of waste as an importance priority value.

*Vip max* denotes the maximum threshold of the importance priority value.

The action is decided by the system by sending the request to the right truck based on the information provided by the truck itself.

**Figure 12:**
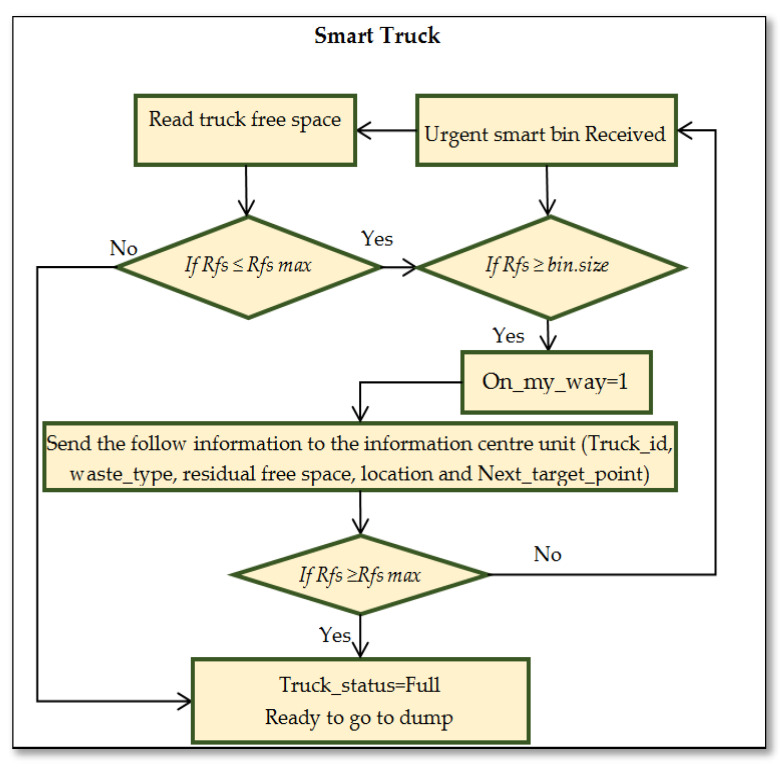
Intelligent smart truck handling information.

Figure 12 presents the flowchart of processing information in the smart truck based on the following conditions:

If Rfs ≤ Rfs max

  {

    Send the urgent notification

    Perform an action

  }

  else

  {

    Read the truck’s residual free space

    Update information

  }

if Vip ≥ Vip max

  {

    Send the information

    Perform an action

  }

  else

  {

    Update information 

  }

where:

*Rfs* denotes the residual available space.

*Rfs max* denotes the maximum threshold value of residual available space.

*Vip* is used for the weight measurement of the danger level of waste as an importance priority value.

*Vip max* denotes the maximum threshold of the importance priority value.

The action is decided by the system to assign the right truck based on the information provided by the truck itself to continue its mission or redirect it to the garbage dump.

Each truck in the zone receives an urgent call message from the full smart bin. The smart truck system checks the available space in the truck, which can include the volume of the collected waste. Then, it sends the information to the central system and mentions if this full smart bin is in the same direction as the truck. Then, the action is decided by the system by sending the request to the right truck based on the information provided by the truck itself. Table 3 presents the parameters that represent the smart truck waste information and its status.

#### 3.2.2. Manual Collection Method

**Figure 13:**
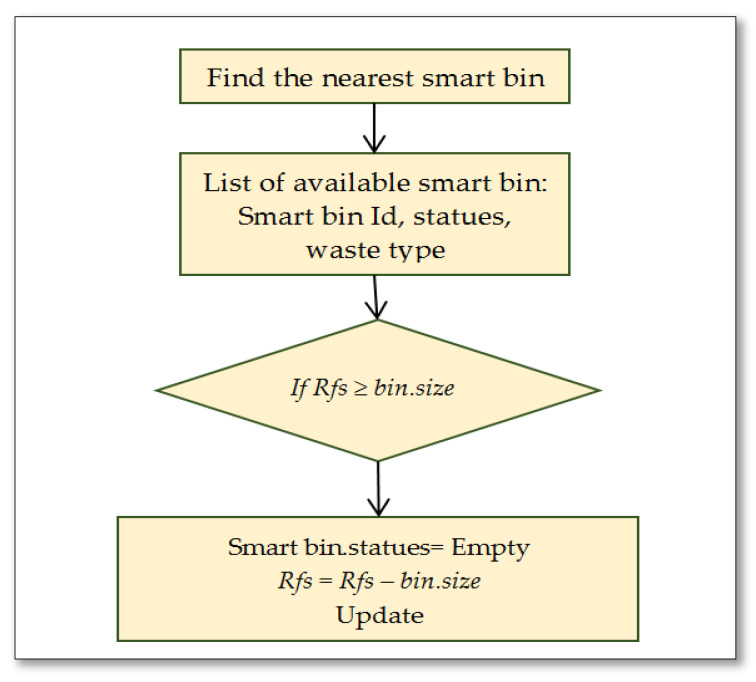
Flowchart of manual collection.

This stage depends on the information provided by the smart bin. The waste collection can be based on a daily schedule or in a frequent manner. The driver can manually select the closed smart bins in the same area that are ready for collection and then update the system, as shown in the flowchart in Figure 13.

### 3.3. Optimization Waste Collection Transportation

The optimization waste collection transportation system is built on simulation. By using heuristic and metaheuristic algorithms used to solve the constraint satisfaction problems, an intelligent waste-gathering transport algorithm is proposed.

Conventional trucks face difficulties in waste collection during peak hours due to the densely inhabited urbanized areas in smart cities, in addition to the historical areas in KSA. This paper proposes having different trucks of dissimilar sizes according to the type of waste and the amount of waste generation in that areas. The small size of trucks can improve traffic congestion and reduce transportation costs, and the trucks can move easily in the area, including the informal and small areas. Trucks are used to collect waste at its source and transport it to the landfill or to the disposal area, where it can be used for renewable energy systems and recycling. Each truck is connected to a GPS/GPRS system to ease the communication between the system entities.

According to the waste type, the proposed waste transportation system uses an efficient algorithm to calculate and find all the smart bins that are subject to be full or will be full soon within the same zoon based on the bin status and find the optimal route towards the urgent bins. The urgent bins are scheduled for waste collection and assigned to the active truck that acts on the request call received from the full smart bin.

The information of each smart bin is retrieved frequently. The location and ID of the target smart bin are received from the MWMC. According to the transportation network and bin distributions, all available paths from the truck origin location to the location of the destination smart bin are calculated by using a genetic algorithm (GA). According to the status of the smart bin for each bin in all available routes, the efficient route is selected.

The decision-making for waste collection for all smart bins is performed by the MWMC according to the available information received from the bins. The efficient route selection is calculated based on the residual bin space of waste, which is the main constraint and the priority importance of collecting waste that reflects the danger of waste as the second constraint parameter.

The transportation network map information is formulated as follows:

Rout (link) *e* (*u*, *v*): *u* ϵ *V*, *v* ϵ *V*, and all link *e*(*u*, *v*) ϵ *E*.

The constraints parameters are as follows:

Residual free space (bin_id) = *R_fs_*(bin_id) < *R_fs_* max: *R_fs_* max is the maximum residual free space in the bin that is considered ready for collection.

Importance of cleaning and danger value (bin_id) = *V_ip_*(bin_id) < *V_ip_* max: *V_ip_* max is the maximum Importance of cleaning and danger value.

The route from the start point location of the truck, which is denoted by S, to the location of the urgent destination bin (T) is defined as a list of smart bins that are labeled as ready to collect.

*R_st_* (S, T) = smartbin_1, smartbin_2, smartbin_3,…, smartbin_*t*,

where all smart bins are on the same route.

*RR_fs_* (S, T) is the total residual free space volume of all smart bins from the starting point of the truck toward the target bin.

*RV_ip_* (S, T) is the route importance of cleaning and danger value for all smart bins from the starting point of the truck towards the target bin.

(1) $$R_{fs}\left(R\left(vs,vt\right)\right)=\sum_{i=s}^{i=t}R_{fs}(\mathrm{bin}\_i):\;\mathrm{bin}\_i\;\epsilon \;R_{\mathrm{st}}\;\mathrm{and}\;1\leq i<n$$

The cost of every available route is computed as follows:

(2) $${RV}_{ip}\left(R\left(vs,vt\right)\right)=\sum_{i=s}^{i=t}V_{ip}(\mathrm{bin}\_i):\;\mathrm{bin}\_i\;\epsilon \;R_{\mathrm{st}}\;\mathrm{and}\;1\leq i<n$$

Based on Equations (1) and (2), the fitness function is calculated as follows:

(3) $$F(R)=Max\;({RV}_{ip}\left(R\left(vs,vt\right)\right),\;R_{fs}\left(R\left(vs,vt\right)\right))$$

The MWMC provides the above-mentioned information according to the received data from smart bins in the municipality area.

In this paper, the proposed algorithm acts on the message received from a certain smart bin that needs a waste collection truck. The algorithm considers all smart bins on the way toward the destination that are going to cross the threshold value of the residual free space rate using GA.

According to the road map and all information provided, the sequence processing of the GA is as follows:

Step1—The population is randomly assigned to one of the available transportation paths based on the bin’s residual statuses. Each path has information on how to deal with the case.

Step2—Assessments: The available transportation paths in a population are assessed by applying the fitness function to identify the top efficient route that includes most of the smart bins with full status.

Step3—Selection: Individuals are selected based on the identified fitness value.

Step4—GA process: The route is converted to enhance the solution.

Step5—Termination: The process ends if the selected route that includes all the smart bins that are ready for collection on the way to the target bin remains the same.

Step6—The population is updated: After route alteration, the inefficient route is avoided and replaced with a new route.

Steps 2, 3, 4, 5 and 6 are repeated until termination.

All locations in the area are concerned in this paper, including the small locations that trucks cannot access. The informal public area is also considered, unlike other researchers and the existing conventional waste system that did not schedule such areas in their work.

**Figure 14:**
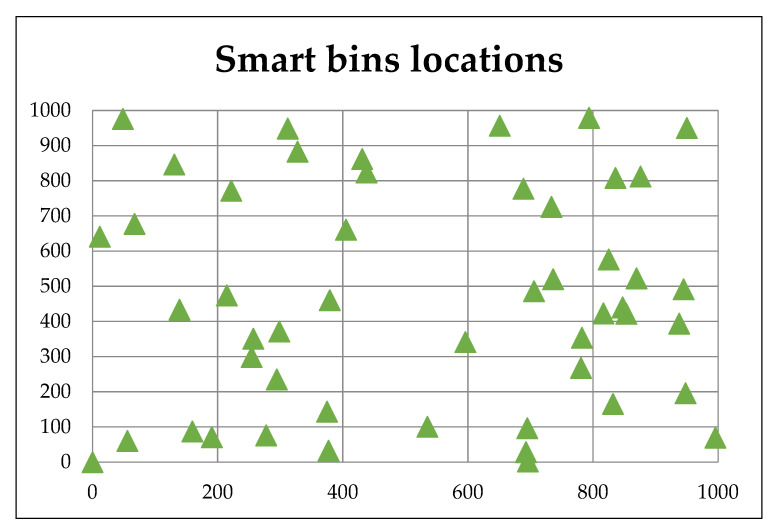
Smart bin location distribution.

In the experiment of this paper, Khalidiyah’s district in Mecca was used as a case study. The area size was assumed to be 1000 m^2^, and the smart bins were randomly distributed, as shown in Figure 14. Each smart bin had a location, status (full/not full/near full) and the expected time it takes to collect the waste from this bin. The experimental evaluation is presented in the experimental results.

### 3.4. Waste Processing

**Figure 15:**
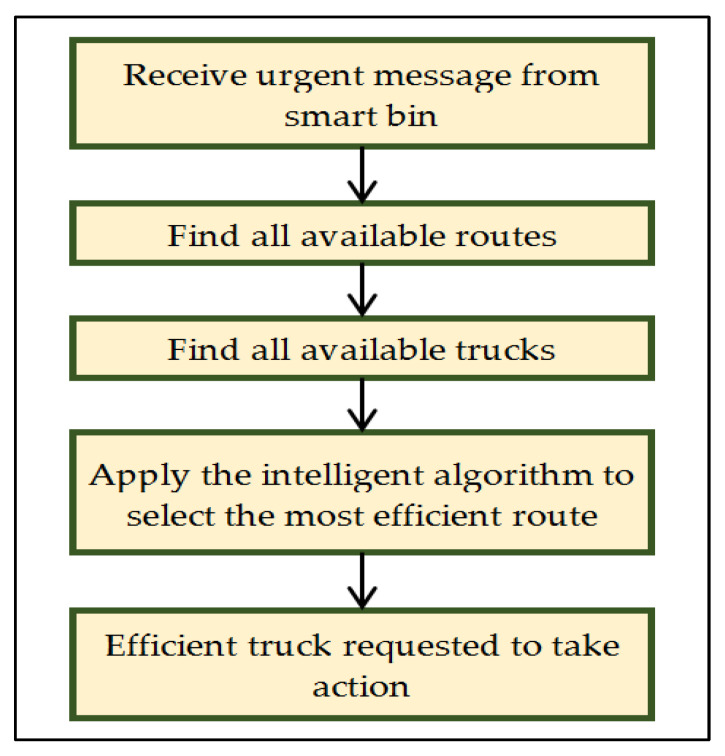
Procedures for decision making.

**Figure 16:**
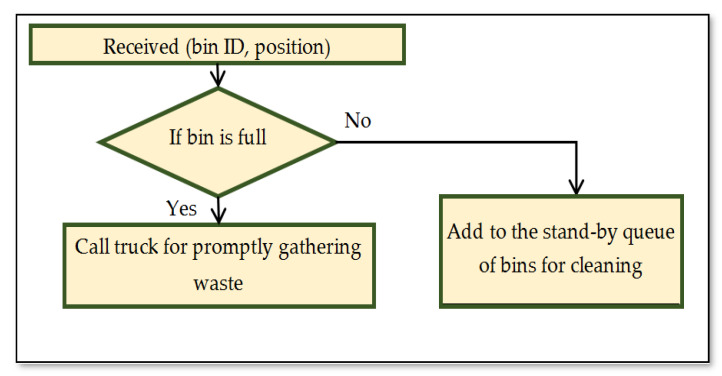
Procedures for decision-making in a smart bin.

The waste management station sends commands to the smart trucks according to the truck location and the information available from the smart bins. Figure 15 and Figure 16 present the proceedings of calling trucks for waste gathering and making decisions. The database of the smart bins is located on a cloud computing infrastructure to supply top data availability. The composite equipment in the bins enables real-time data gathering. The waste management station analyses the assembled information from the IoT equipment and supplies helpful data for decision-making inside the intelligent waste management system.

## 4. Evaluation Results

Evaluation is carried out through three scenarios. Each scenario uses a single truck applied for one waste type towards the 50 locations. The experiment is simulated and executed utilizing C++ and MATLAB by producing a random distribution for smart bins amongst 50 locations on 1000 m^2^ as a part of the Khalidiyah zone and waste information, including smart bin status and waste type. The case values are randomly appointed for each smart bin to ensure conformance. Amongst all available routes to the smart bins, the most efficient routes are calculated on the grounds of the GA. Various routes are assessed to locate the most efficient route from the start point of smart trucks to the urgent smart bins that require cleaning.

In this paper, assessment experiments are implemented for three smart trucks assigned to collect the waste from the sorted urgent smart bin based on waste type and other smart bins considered ready for collection from the same waste type.

**Figure 17:**
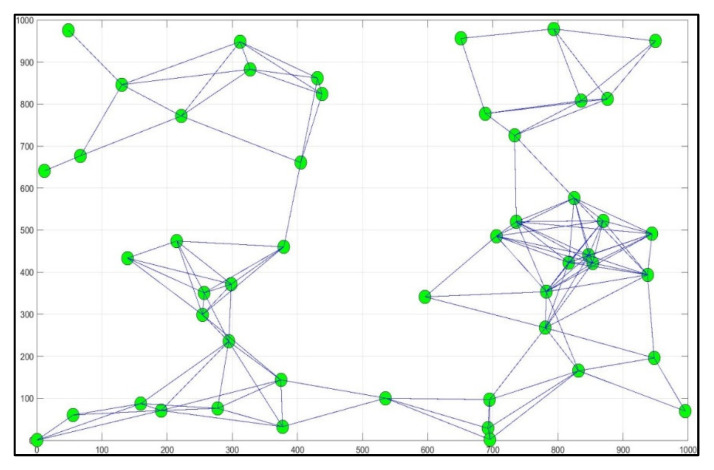
Possible routes between all locations.

All public areas and the other residents’ service locations are considered in this experiment, with no specific location to be cleaned more than any other. Unlike the existing system, priority is assigned depending on the smart bin waste status and waste source of public service places, such as supermarkets, homes, retail, restaurants and hospitals, as well as the risk of the waste and the importance of the place. Figure 17 shows the random locations of the smart bins amongst the site and the possible routes between all locations.

**Figure 18:**
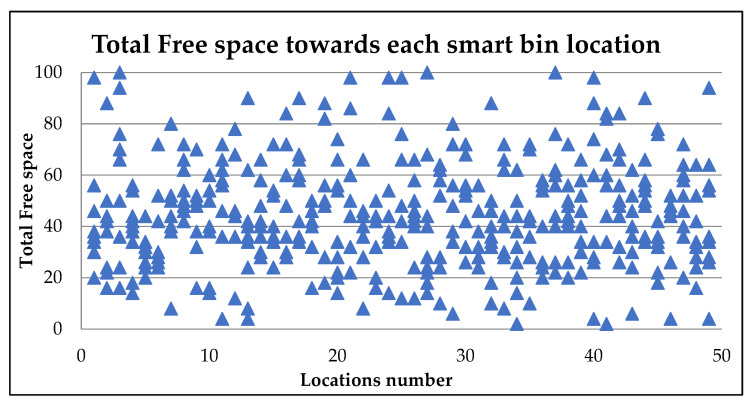
Total free space towards each smart bin location-Type 1.

Figure 18 presents the calculated total free space for each available route to every smart bin in the area. Numbers of smart bins below 10% of the total free space are considered ready for collection and may call for urgent cleaning. The truck collects the waste from all the smart bins that are closed to be ready for collection in the same way as the urgent smart bin that needs immediate cleaning that has around 20% free space. Table 4 presents the urgent smart bin, including the other smart bins the subject to be full soon and the other bins near the threshold point of 20% free space. This action reduces the consumption of time for waste transport collection on the same day and the next day as well. Moreover, these locations include free public areas and other unseen locations considered in this research as public service locations. Figure 18 shows the total free space for each location has different reading values according to the frequent reading time and the updated information. After the free space percentage becomes less than 10%, the smart bin keeps updating the central server for any increase in the waste until the truck arrives and changes the status of free space to 100%.

**Figure 19:**
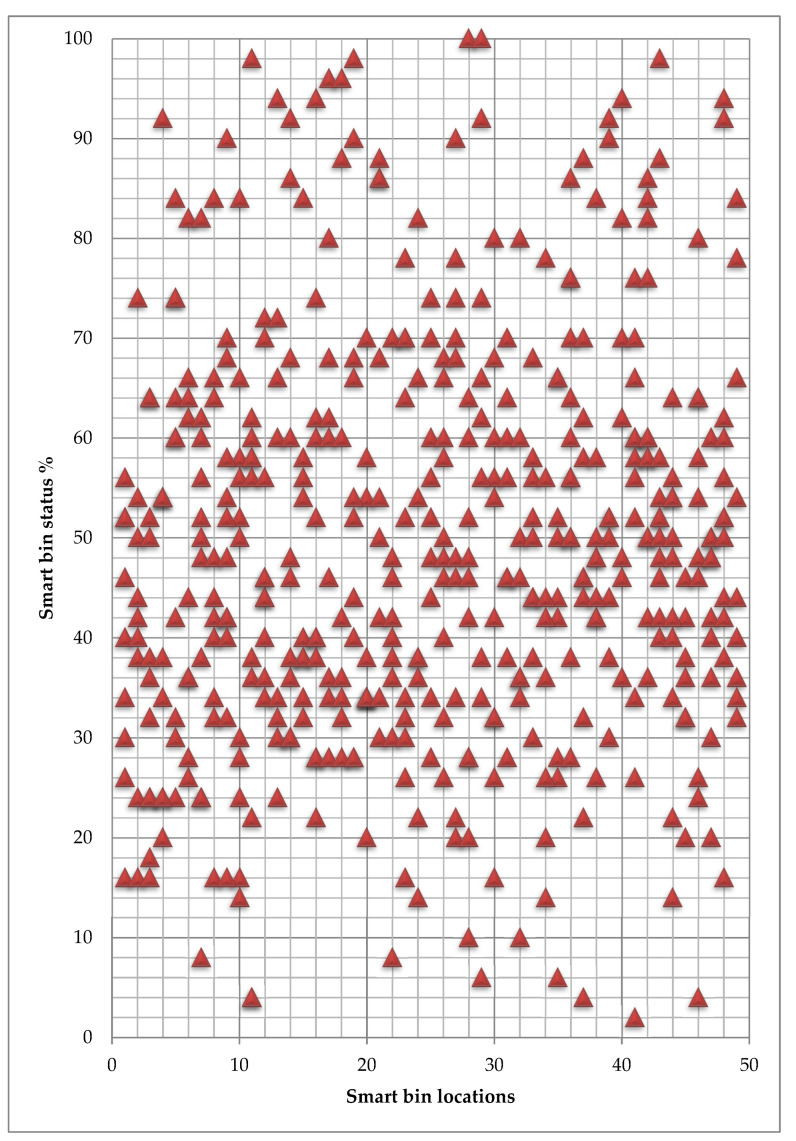
Smart bin status towards each smart bin location-Type 2.

**Figure 20:**
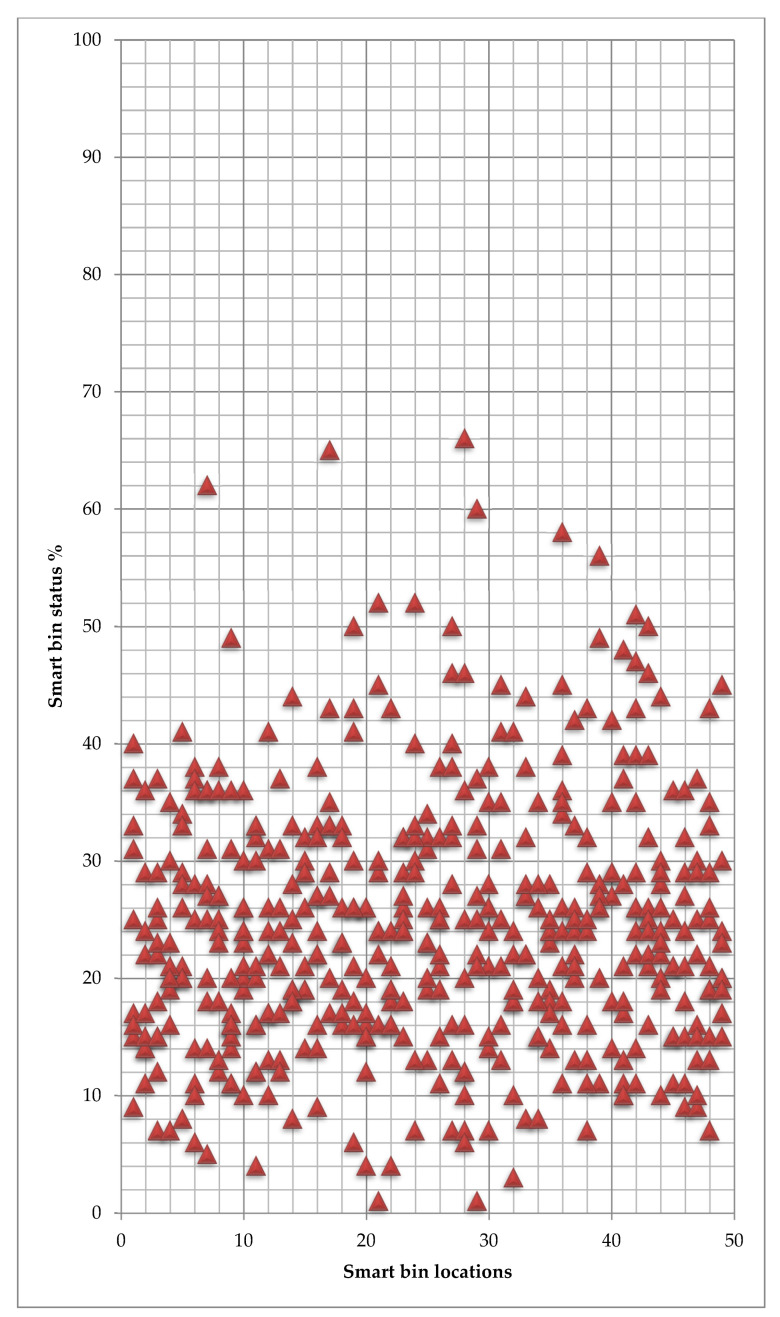
Smart bin status towards each smart bin location-Type 3.

Figure 19 and Figure 20 present the average waste volume for each available route to every smart bin in the area for waste types 2 and 3, respectively. The numbers of smart bins reach 10% of the total free space, are considered ready for collection and may call for urgent cleaning.

The truck collects the waste from all the smart bins that are close to being ready for collection in the same way as the urgent smart bin that needs immediate cleaning that has around 20% of free space. Table 5 presents the urgent smart bin waste status, including the other smart bins the subject to be full soon and the other bins near the point of 80% of waste volume. This action also reduces the consumption of time for waste transport collection on the same day and the next day as well for the waste type.

Figure 20 clearly shows no smart bin is ready for collection. All smart bins in the spot area have not reached the threshold value to be ready for collection.

## 5. Comparison and Discussion

The waste recycling system aims to collect the waste based on the waste type to improve the recycling management system and provide a healthy environment in addition to optimizing route transportation of waste gathering, according to GA.

The results show the number of sites of smart bins that require cleaning, including the other smart bins in the calculated route variant between Types 1 and 2, whereas Type 3 does not have any smart bins ready for collection. The simulation considers the different types of waste that have a different percentage of waste generation based on the waste source location.

**Figure 21:**
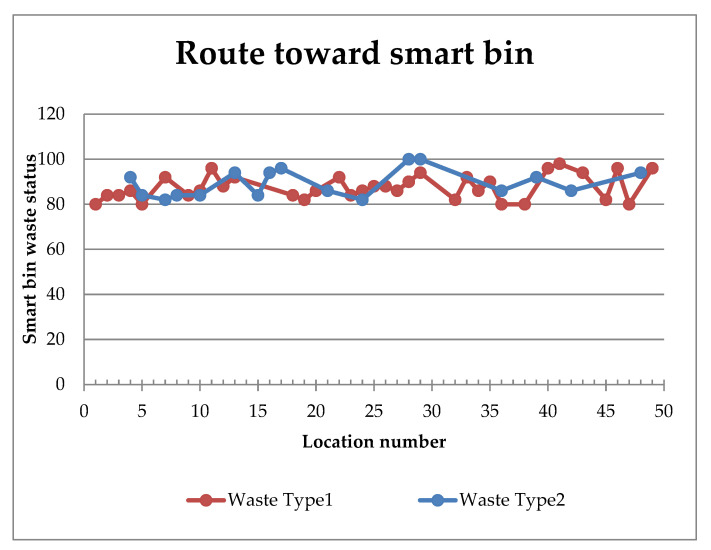
The route towards the smart bin.

The locations towards the urgent smart bin are calculated based on the GA. These locations include most of the urgent smart bins. The smart truck starts from the municipality center through the locations towards the urgent smart bin and collects the waste in the same direction. The urgent smart bins listed in the locations towards the target bin are skipped from the next truck travel for collection as the bin is already clean. Thus, after filtering the urgent bins from the collected bins, the number of locations to visit from the list of urgent smart bins is reduced, which results in reduced time consumption too. Table 6 presents the urgent smart bins locations that need to be cleaned from Type 2. Figure 21 presents the calculated route toward the smart bin for waste Types 1 and 2. Thus, this paper will help waste recycling organizations to maintain waste collection transportation based on the waste type.

In case the company provides a conventional truck for all waste types or the container in the truck is divided into different parts to carry different waste types, the system can combine urgent smart locations in one list and use the route toward the urgent smart bin. Also, the driver of the truck can select the target smart bin based on his location manually, as mentioned in the strategy of waste collection. Moreover, from the literature above, it is clear most of the previous systems proposed to manage solid waste, even when they are sorted and perform isolation for recycling, did not mention how they collect from underground containers.

The proposed method focused on the population density and public services locations, in addition to the waste type. That earns more points compared to the existing methods. In comparison to the proposed method by [23], our study focused on the source waste locations and the public service locations, including the open public areas, in addition to the waste type identification, which is related to the waste source location. The proposed method outperforms the [23] method that is only concerned with the landfill area, which is the last stage in the waste management system. The isolation of waste in the early stage from the source location improves the recycling system and healthy environment by collecting the waste at the right time for each waste type.

On the other hand, Makkah-SWMS, which is proposed by [30], splits up the waste into five kinds and transmits a notification message to the monitoring system when the trash bin is overloaded. This method receives the waste from one input which is the trash can itself, and then the separation in the next step. The alert message will be sent if the trash can is full of any type. That will result in a waste of time and high costs of transportation and waste collection compared to the proposed method that provides different smart bins for each waste type, and only the right truck will come at the right time.

## 6. Conclusions

In smart city applications, a pressing issue is to find a way to manipulate waste with minimum expense and superior results. Waste is detrimental to the goal of a smart metropolis that is very satisfying to society. Mecca and the holy sites are in the same region. Arafat, Mina and Muzdalifah are densely populated zones where waste disposal is a major problem. Four elements make it a great mission, the natural area, the small space, the time and the growth of Pilgrim members and population. The technique of collecting waste, separating it and transporting the boxes rapidly and daily to keep away any prospect of various diseases is a complicated technique. The case study area is not set up as a smart city yet and still uses the conventional waste transportation system with a predetermined route map. This paper’s goal is to address the idea of waste control and smart structural proposals for waste collection. The proposed system simulates IoT in the container and trucks that secure the overflow and separation of waste. Waste source locations and population density affect the volume of waste generation, especially waste food, as it is the highest in terms of waste generation. The open public areas and the small space location problem is solved by the proposed different truck sizes based on the waste type. Each container is used for one type of waste, such as food, plastic and others, and uses the optimization algorithm to calculate and find the optimal route toward the full waste container. The proposed system outperforms other systems by reducing the number of locations and smart bins that have to be visited by 20% for Type 1 and 18% for Type 2, whereas the conventional and existing system has to visit all locations every day, which results in high cost and time consumption. Moreover, according to the last smart bin urgent call for Types 1 and 2, all the smart bins of Type 3 are not considered ready for collection, which reduces the transportation cost and time consumption by 100% for this day. Most of the studies are facing limitations when it comes to the implementation stage. The main limitation that was faced at the work stage was the difficulty of installing the smart devices in the system components due to missing network infrastructure in the city. These things were not widely available. Hence, the second choice to be done is to simulate and present how this proposed system provides a smart solution for the actual city to get the advantages among the economy, environment, and humans. All the information presented in this study could be presented to the companies that wish to build smart infrastructure in the future when the capabilities become available. However, expanding the area of study with more density and analyzing the information over all the districts and regions of KSA will get more valuable data that can improve the environment and health suitability.

## Figures and Tables

**Table 1:** Waste resource in the al-Misfalah district part of Mecca.

District/حي	Groceries	Bakeries	Restaurants	Hospitals	Pharmacies
**Al-Misfalah المسفلة**	57	8	31	1	5
**Al-Rusifia الرصيفية**	32	3	32	4	8
**Al-Khaldiyeh الخالدية**	50	3	25	10	15
**Al-Taqwa التقوى**	28	1	17	0	8
**Al-Hindawiyah الهنداوية**	35	2	16	1	4
**Al-Rawaby الروابي**	7	0	6	0	0
**KDE كدي**	14	3	10	1	2

**Table 2:** Parameters of smart bin waste information.

Parameter	Description
**Bin_id**	Every smart bin has a unique ID, including the district area
**waste_type**	For sorted waste (Organic, Solid, Liquid, etc.)
**residual free space**	The free space in the container
**location**	the location through GPS
**waste priority value**	The danger of waste has high priority

**Table 3:** Parameters of smart truck waste information.

Parameter	Description
**Truck_id**	Every smart bin has a unique ID, including the district area
**waste_type**	For sorted waste (Organic, Solid, Liquid, etc.)
**residual free space**	The free space in the container
**location**	the location through GPS
**waste priority value**	The danger of waste has high priority
**Next_target_point**	The location of the next smart bin that needs cleaning

**Table 4:** Urgent smart bins needed to be cleaned-Type 1.

Smart Bin Number	Locations toward Smart Bin	Average of Total Free Space
**1**	48 1	20
**2**	15 2	16
**3**	48 3	16
**4**	37 4	14
**5**	37 28 5	20
**7**	15 7	8
**9**	14 9	16
**10**	14 10	14
**11**	31 11	4
**12**	37 25 12	12
**13**	15 13	8
**18**	48 18	16
**19**	48 19	18
**20**	14 20	14
**22**	14 22	8
**23**	31 23	16
**24**	14 24	14
**25**	37 25	12
**26**	26	12
**27**	14 27	14
**28**	15 28	10
**29**	37 29	6
**32**	32	18
**33**	14 33	8
**34**	15 34	14
**35**	15 35	10
**36**	34 36	20
**38**	48 18 38	20
**40**	31 40	4
**41**	15 41	2
**43**	31 43	6
**45**	48 45	18
**46**	37 46	4
**47**	15 47	20
**48**	48	16
**49**	15 49	4
**Total number of smart bins in need of urgent cleaning**		36

**Table 5:** Urgent smart bins needed to be cleaned-Type 2.

Smart Bin Number	Locations toward Smart Bin	Smart Bin Waste Status
**28**	19 20 30 14 28	100
**29**	11 40 43 29	100
**11**	23 38 18 16 26 11	98
**19**	29 8 37 19	98
**43**	14 20 13 5 45 43	98
**17**	6 49 18 42 17	96
**18**	38 35 21 18	96
**13**	35 12 13	94
**16**	21 18 3 16	94
**40**	35 14 38 40	94
**48**	21 30 48	94
**4**	32 12 27 4	92
**14**	29 43 31 14	92
**39**	37 22 30 9 39	92
**9**	26 40 2 9	90
**27**	29 33 4 27	90
**21**	26 42 10 6 18 21	88
**37**	48 34 3 37	88
**21**	48 41 46 21	86
**36**	27 41 36	86
**42**	18 25 12 42	86
**5**	6 48 1 5	84
**8**	38 17 36 8	84
**10**	38 40 10	84
**15**	16 36 15	84
**38**	11 4 1 38	84
**49**	20 10 49	84
**6**	18 10 6	82
**7**	35 37 3 7	82
**24**	14 20 47 24	82
**30**	49 3 7 21 30	80
**32**	27 14 38 32	80
**46**	16 24 5 46	80
**Total number of routes toward the urgent bins**		33

**Table 6:** Routes to urgent smart bin locations-Type 2.

Smart Bin Number	Locations toward Smart Bin	Smart Bin Waste Status
**28**	19 20 30 14 28	100
**29**	11 40 43 29	100
**17**	6 49 18 42 17	96
**13**	35 12 13	94
**16**	21 18 3 16	94
**48**	21 30 48	94
**4**	32 12 27 4	92
**39**	37 22 30 9 39	92
**21**	48 41 46 21	86
**36**	27 41 36	86
**42**	18 25 12 42	86
**5**	6 48 1 5	84
**8**	38 17 36 8	84
**10**	38 40 10	84
**15**	16 36 15	84
**7**	35 37 3 7	82
**24**	14 20 47 24	82
**Total number of routes to toward the urgent bins**		17

## References

[1] Nizami A., Rehan M., Naqvi M., Ouda O., Shahzad K., Syamsiro M., Waqas M., Miandad R., Asam Z., Ismail I.M. (2017). Energy, economic and environmental savings by waste recycling: A case study of Madinah city. Energy Procedia.

[2] Shambour M.K., Gutub A. (2022). Progress of IoT research technologies and applications serving Hajj and Umrah. Arab. J. Sci. Eng..

[3] Rehan M., Nizami A.S., Shahzad K., Ouda O.K.M., Ismail I.M.I., Almeelbi T., Iqbal T., Demirbas A. (2016). Pyrolytic liquid fuel: A source of renewable electricity generation in Makkah. Energy Sources Part A Recovery Util. Environ. Eff..

[4] Abdullah N., Al-Wesabi O.A., Mohammed B.A., Al-Mekhlafi Z.G., Alazmi M., Alsaffar M., Sumari P. (2022). Integrated Approach to Achieve a Sustainable Organic Waste Management System in Saudi Arabia. Foods.

[5] Osra F.A., Osra K.A., Jones P., Osra O.A., Suhartini N., Alzahrani J.S., Mirza A.Z. (2022). Public Space and Solid Waste Facilities in Makkah. Arab. J. Geosci..

[6] Majrashi A. (2017). Development-induced displacement of informal settlements in Makkah, Saudi Arabia. Ph.D. Thesis.

[7] Derbali A., Farhi A. (2022). Demographic Analysis of the Makkah Province for the Purpose of Evaluating the Balance of the Urban System. Quaest. Geogr..

[8] Bayounis A., Eldamaty T. (2022). Applying Geographic Information System (GIS) for Solar Power Plants Site Selection Support in Makkah. Technol. Invest..

[9] Lewis-Lettington R., El-Hefnawi A., Bajaj M. (2019). Makkah City Profile. UN-Habitat.

[10] (2017). The 16th Services Guide for Makkah Al Mukarramah Region, Grity for Statistics. https://www.stats.gov.sa/sites/default/files/makkah_al-mokarramah_region_en.pdf.

[11] Bagnied H., El-Damaty T.A. (2022). Scientia Research Library ISSN 2348-0424. J. Eng. Technol. Res..

[12] Saudi Press Agency The Landfill of the Holy Capital Municipality Receives More Than 68 Thousand Tons of Waste. https://www.spa.gov.sa/2265087/.

[13] Saudi Press Agency 37% of the Waste Wasted in Makkah is “Organic”. https://www.spa.gov.sa/2219953/.

[14] Alharbi A.B. (2015). Native Settlements in Makkah Al-Mukarramah Area and Factors Affecting Its Distribution. Adv. Anthropol..

[15] Daiem M.M.A., Said N. (2022). Energetic, economic, and environmental perspectives of power generation from residual biomass in Saudi Arabia. Alex. Eng. J..

[16] Opazo Basaez M., Ghulam Muhammad S., Arias Aranda D., Molina Moreno V. (2015). A roadmap towards smart services in healthcare. Dyna.

[17] Adedeji K.B., Ponnle A.A., Abu-Mahfouz A.M., Kurien A.M. (2022). Towards Digitalization of Water Supply Systems for Sustainable Smart City Development—Water 4.0. Appl. Sci..

[18] Wang J., Liu C., Zhou L., Xu J., Wang J., Sang Z. (2022). Progress of Standardization of Urban Infrastructure in Smart City. Standards.

[19] The World Bank Group Trends in Solid Waste Management. https://datatopics.worldbank.org/what-a-waste/trends_in_solid_waste_management.html/.

[20] Chen D.M.C., Bodirsky B.L., Krueger T., Mishra A., Popp A. (2020). The world’s growing municipal solid waste: Trends and impacts. Environ. Res. Lett..

[21] UN Environment Programme (UNEP) UNEP Food Waste Index Report 2021. https://www.unep.org/resources/report/unep-food-waste-index-report-2021/.

[22] Yaagoubi R., Faisal K., Miky Y. (2021). Land value assessment based on an integrated Voronoi-geographically weighted regression approach in Makkah, Saudi Arabia. J. King Saud. Univ. Sci..

[23] Baig M.B., Gorski I., Neff R.A. (2019). Understanding and addressing waste of food in the Kingdom of Saudi Arabia. Saudi J. Biol. Sci..

[24] Osra F.A., Ozcan H.K., Alzahrani J.S., Alsoufi M.S. (2021). Municipal solid waste characterization and landfill gas generation in kakia landfill, makkah. Sustainability.

[25] Ali A.M., Nawaz A.M., Al-Turaif H.A., Shahzad K. (2021). The economic and environmental analysis of energy production from slaughterhouse waste in Saudi Arabia. Environ. Dev. Sustain..

[26] Ouda O.K., Raza S.A., Al-Waked R., Al-Asad J.F., Nizami A.S. (2017). Waste-to-energy potential in the Western Province of Saudi Arabia. J. King Saud. Univ. Eng. Sci..

[27] EcoMENA Solid Waste Management in Saudi Arabia. https://www.ecomena.org/solid-waste-management-in-saudi-arabia/.

[28] Abdullah N., Alwesabi O.A., Abdullah R., Saeed F., Gazem N., Mohammed F., Busalim A. (2019). IoT-Based Smart Waste Management System in a Smart City. Recent Trends in Data Science and Soft Computing, Advances in Intelligent Systems and Computing.

[29] MetroTaifun Mecca in Saudi Arabia. http://www.metrotaifun.com/automatic_solid_waste_collection_system/en/references/selected-references/724-mecca-reference.html.

[30] Elhassan R., Ahmed M.A., AbdAlhalem R. Smart waste management system for crowded area: Makkah and holy sites as a model. Proceedings of the 4th MEC International Conference on Big Data and Smart City (ICBDSC), IEEE.

[31] Dini I. (2021). Bio Discarded from Waste to Resource. Foods.

[32] Berry J.B., Otley D.T., Lee B.C. (2004). Case-Based Research in Accounting. The Real Life Guide to Accounting Research.

[33] Creswell J.W., Creswell J.D. (2017). Research Design: Qualitative, Quantitative, and Mixed Methods Approaches.

[34] Neuman W.L., Robson K. (2014). Basics of Social Research.

[35] Thornhill A., Saunders M., Lewis P. (2009). Research Methods for Business Students.

[36] Wahyuni D. (2012). The research design maze: Understanding paradigms, cases, methods and methodologies. J. Appl. Manag. Account. Res..

[37] Woodside A.G. (2010). Case Study Research: Theory, Methods and Practice.

[38] Yin R.K. (2012). Applications of Case Study Research.

[39] Chaudhari M.S., Patil B., Raut V. Iot based waste collection management system for smart cities: An overview. Proceedings of the 3rd International Conference on Computing Methodologies and Communication (ICCMC).

[40] Gupta P.K., Shree V., Hiremath L., Rajendran S. (2019). The Use of Modern Technology in Smart Waste Management and Recycling: Artificial Intelligence and Machine Learning. Studies in Computational Intelligence.

[41] Anagnostopoulos T., Zaslavsky A., Kolomvatsos K., Medvedev A., Amirian P., Morley J., Hadjieftymiades S. (2017). Challenges and Opportunities of Waste Management in IoT-Enabled Smart Cities: A Survey. IEEE Trans. Sustain. Comput..

[42] Vitorino de Souza Melaré A., Montenegro González S., Faceli K., Casadei V. (2017). Technologies and decision support systems to aid solid-waste management: A systematic review. Waste Manag..

[43] Abdullah N., Al-wesabi O.A., Mohammed B.A., Al-Mekhlafi Z.G., Alazmi M., Alsaffar M.S., Sumari P. (2021). Improving waste management system efficiency and mobility with efficient path MANET. Appl. Sci..

[44] Mak T.M.W., Xiong X., Tsang D.C.W., Yu I.K.M., Sun Poon C. (2019). Sustainable food waste management towards circular bioeconomy: Policy review, limitations and opportunities. Bioresour. Technol..

[45] Lai C.F., Lai Y.X., Yang L.T., Chao H.C. Integration of IoT energy management system with appliance and activity recognition. Proceedings of the CPSCom 2012.

[46] Ramallo-González A.P., Bardaki C., Kotsopoulos D., Tomat V., González Vidal A., Fernandez Ruiz P.J., Skarmeta Gómez A. (2022). Reducing Energy Consumption in the Workplace via IoT-Allowed Behavioural Change Interventions. Buildings.

[47] Papaioannou T., Dimitriou N., Vasilakis K., Garbi A. (2018). An IoT-Based Gamified Approach for Reducing Occupants’ Energy Wastage in Public Buildings. Sensors.

[48] Hermsen S., Frost J., Renes R.J., Kerkhof P. (2016). Using feedback through digital technology to disrupt and change habitual behavior: A critical review of current literature. Comput. Hum. Behav..

[49] Anagnostopoulos T., Kolomvatsos K., Anagnostopoulos C., Zaslavsky A., Hadjiefthymiades S. (2015). Assessing dynamic models for high priority waste collection in smart cities. J. Syst. Softw..

[50] Cerchecci M., Luti F., Mecocci A., Parrino S., Peruzzi G., Pozzebon A. (2018). A low power IoT sensor node architecture for waste management within smart cities context. Sensors.

[51] Navghane S.S., Killedar M.S., Rohokale V.M. (2016). IoT based smart garbage and waste collection bin. Int. J. Adv. Res. Electron. Commun. Eng..

[52] Medvedev A., Fedchenkov P., Zaslavsky A., Anagnostopoulos T., Khoruzhnikov S., Balandin S., Andreev S., Koucheryavy Y. (2015). Waste management as an IoT-enabled service in smart cities. Internet of Things, Smart Spaces and Next Generation Networks and Systems.

[53] Hassan H., Saad F., Fazlin N., Aziz A. Waste monitoring system based on Internet-of-thing (IoT). Proceedings of the IEEE Conference on Systems, Process and Control (ICSPC).

